# Comparing folded-flap palatoplasty and staphylectomy in brachycephalic dogs: complications and discharge time

**DOI:** 10.18849/ve.v10i3.716

**Published:** 2025-09-15

**Authors:** Mohamed Sherif+

**Keywords:** AIRWAY SURGERY, BOAS, BRACHYCEPHALIC OBSTRUCTIVE AIRWAY SYNDROME, ELONGATED SOFT PALATE, FOLDED FLAP PALATOPLASTY, STAPHYLECTOMY

## Abstract

**PICO question:**

In dogs undergoing brachycephalic obstructive airway syndrome (BOAS) surgery, is folded-flap palatoplasty associated with fewer complications and earlier discharge time compared with staphylectomy techniques?

**Clinical bottom line:**

**Category of research:**

Risk.

**Number and type of study designs reviewed:**

Two retrospective cohort studies compared outcomes of folded-flap palatoplasty and staphylectomy. One study performed staphylectomy using the standard cut-and-sew technique, while the other used a CO_2_ laser. Folded-flap palatoplasty was carried out using the standard technique described in the literature in both studies.

**Strength of evidence:**

Weak.

**Outcomes reported:**

While one study reports similar outcomes in minor and major complications between folded-flap palatoplasty and staphylectomy, with both procedures having comparable hospitalisation durations, another study identifies staphylectomy as one of four factors associated with a complicated recovery, along with increasing age, higher grades of laryngeal collapse, and prolonged anaesthesia time.

**Conclusion:**

To date, there is weak evidence suggesting that staphylectomy may be one of the risk factors for perioperative complications in dogs undergoing surgery for an elongated soft palate. This potential risk should be considered during surgical planning and immediate postoperative period.

## How to apply this evidence in practice

The application of evidence into practice should take into account multiple factors, not limited to: individual clinical expertise, patient’s circumstances and owners’ values, country, location or clinic where you work, the individual case in front of you, the availability of therapies and resources.

Knowledge Summaries are a resource to help reinforce or inform decision-making. They do not override the responsibility or judgement of the practitioner to do what is best for the animal in their care.

## Clinical scenario

You are presented with a brachycephalic dog for assessment of stertorous breathing, exercise intolerance, and occasional regurgitation. Your physical examination revealed stenotic nares and an airway examination revealed an elongated soft palate. You are familiar with the surgical techniques for elongated soft palate resection including folded-flap palatoplasty and staphylectomy. While you are well-versed in the conventional staphylectomy technique, your experience with folded-flap palatoplasty is limited, necessitating a referral to a specialist. You need to discuss the current evidence with the owners to help them decide which procedure they would like to proceed with.

## The evidence

The current evidence favouring folded-flap palatoplasty over staphylectomy is weak. Two retrospective cohort studies (Fracka et al., 2024; Miller et al., 2024) directly compared both surgical techniques. Retrospective studies come with inherent biases and offer less robust evidence when compared with prospective studies.

Miller et al. (2024) concluded that staphylectomy and folded-flap palatoplasty showed similar perioperative complication rates. However, the assessment did not account for the use of vessel-sealing devices, electrocautery, or laser. Additionally, no preoperative measurement of soft palate thickness – which could affect the choice of surgical technique – was performed. The low prevalence of clinical signs suggested that Brachycephalic Obstructive Airway Syndrome (BOAS) surgery was often conducted as a preventative measure. The small sample size may have also influenced the results, particularly the complication rate, potentially leading to a type II statistical error.

Fracka et al. (2024) identified staphylectomy as one of four variables associated with complicated recovery in dogs undergoing upper airway surgery. Other factors included increasing age, a higher grade of laryngeal collapse, and longer durations of general anaesthesia. However, like the first study (Miller et al., 2024), this one was also retrospective, carrying the same inherent limitations. A small sample size and the low incidence of complicated recovery may have introduced an upward bias, requiring cautious interpretation of the results. Additionally, the folded-flap palatoplasty procedure was not standardised, as it involved a mix of sharp dissection, CO_2_ laser, and bipolar cautery techniques.

As a result, it is difficult to recommend one surgical technique over the other. There is weak evidence suggesting that staphylectomy is one of the variables associated with a complicated recovery in dogs undergoing upper airway surgery. Further prospective studies comparing staphylectomy techniques with folded-flap palatoplasty are needed.

## Summary of the evidence

**Fracka et al. (2024):** Risk factors for complicated perioperative recovery in dogs undergoing staphylectomy or folded flap palatoplasty: Seventy-six cases (2018–2022) **Aim: **To identify the risk factors for complicated preoperative recovery in dogs undergoing either staphylectomy or folded-flap palatoplasty for brachycephalic obstructive airway syndrome (BOAS) corrective upper airway surgery.

Population:	Client owned dogs who underwent upper airway surgery for brachycephalic obstructive airway syndrome (BOAS) between January 2018 and July 2022.
Sample size:	76 dogs.
Intervention details:	Median age was 25 months, and the median weight was 11 kg. The most common breed was Bulldogs 61/76 (80%). All 76 dogs had been diagnosed with elongated soft palate and underwent either staphylectomy (39/76) or folded-flap palatoplasty (37/76). All the staphylectomy procedures were performed using CO_2_ Of the 37 dogs that underwent folded-flap palatoplasty, 23 dogs had the procedure performed with CO_2_ laser, 9 dogs underwent sharp dissection using Metzenbaum scissors and bipolar electrocautery for haemostasis, and 5 dogs had a combination of sharp dissection and laser treatment.
Study design:	Retrospective cohort study.
Outcome Studied:	The studies reported outcomes including reason for presentation, clinical findings on admission, need for oxygen or sedation, relevant history, diagnostic test results, and the presence and grade of laryngeal collapse or paralysis. A retrospective brachycephalic risk (BRisk) score was also calculated. Intraoperative outcomes assessed included anaesthetic and surgical times, type of BOAS procedure performed, additional concurrent procedures, and any respiratory or cardiovascular complications. Postoperative outcomes included oxygen supplementation, use of dexmedetomidine infusion, tracheostomy, duration of hospitalisation, medications and diet provided, and mortality during hospitalisation. Complications: Complications were categorised as respiratory or gastrointestinal. Minor: oxygen support for less than 12 hours, regurgitation, and nausea. Major: dyspnoea, oxygen support for more than 12 hours, aspiration pneumonia, the need for temporary tracheostomy, sedation, intubation, or death prior to discharge. Complicated recovery was defined as oxygen requirement of more than 12 hours, and/or the need for temporary tracheostomy, or death.
Main Findings (relevant to PICO question):	31/76 dogs (41%) had additional procedures performed under the same anaesthetic event. Of these dogs 26/31 had surgical procedures and 5/31 had nonsurgical procedures. Postoperative complications: 30/76 dogs experienced postoperative complications. 16 dogs had major complications, and 14 dogs had minor complications. All 16 dogs with major complications were either requiring prolonged oxygen treatment of more than 12 hours and/or needed a tracheotomy and/or died during recovery. Of the 16 dogs that experienced a complicated recovery, 11 had undergone staphylectomy and 5 had undergone folded-flap palatoplasty. When included in the multivariable analysis, factors including staphylectomy surgery, increasing age (median age of complicated recovery dogs was 74 months), higher laryngeal collapse grade of > 2 (In grade 2 laryngeal collapse, the cuneiform process of the arytenoid cartilage loses its rigidity and becomes medially displaced), and longer duration of general anaesthesia were associated with complicated recovery in dogs undergoing staphylectomy or folded-flap palatoplasty. However, in the univariable analysis, the type of surgery was not linked to the outcome. Hospitalisation: Median duration of hospitalisation postoperatively was 1.5 days. 73/76 dogs (96%) survived to discharge. Three dogs were euthanised 1–2 days following airway surgery for persistent severe dyspnoea. The paper didn’t mention the type of airway surgery performed on the dogs that were euthanised.
Limitations:	Medical records were not standardised, and some information, such as the description of clinical signs and perioperative complications, may be incomplete. Laryngeal evaluations were conducted by different clinicians with varying levels of experience, making standardisation impossible. There was no standardised postoperative protocol, as treatment decisions were based on individual clinician preferences. The limited sample size may have affected the statistical analysis and increased the risk of type II error, while the rare occurrence of complicated recovery may have introduced an upward bias.

**Miller et al. (2024):** Complications and outcome following staphylectomy and folded flap palatoplasty in dogs with brachycephalic obstructive airway syndrome **Aim: **To compare the prevalence of preoperative, intraoperative, and postoperative variables and complications associated with staphylectomy and folded-flap palatoplasty in dogs undergoing corrective upper airway surgery for brachycephalic obstructive airway syndrome (BOAS).

Population:	Client owned dogs who underwent surgical treatment for elongated soft palate at the University of Florida Small Animal Veterinary Hospital (USA) between 2012 and 2019. The three most common breeds were French Bulldog, English Bulldog, and Pug.
Sample size:	124 dogs.
Intervention details:	Medical records were retrospectively reviewed for dogs that underwent staphylectomy or folded-flap palatoplasty. The records do not indicate whether the choice of surgical intervention was based on the surgeon’s preference or the dog’s condition. 64/124 underwent staphylectomy. 60/124 underwent folded-flap palatoplasty. Staphylectomy was performed using a traditional cut-and-sew technique. No vessel-sealing device, electrocautery, or laser were used. Folded-flap palatoplasty was performed as described in the literature. Dogs were excluded if they had; incomplete medical records. diagnosis of laryngeal paralysis, concurrent airway surgeries that are unrelated to brachycephalic obstructive airway syndrome (BOAS) (i.e., cleft palate), or endoscopically performed soft palate surgery.
Study design:	Retrospective cohort study.
Outcome Studied:	The studies reported outcomes including perioperative complications, anaesthetic time, concurrent surgical procedures, length of hospitalisation, requirement for oxygen supplementation, postoperative medication use, and the incidence of regurgitation or aspiration pneumonia. Complications: Minor: did not require additional surgical management and/or did not lead to death within two weeks of surgery. Major: additional surgical management was required and/or death occurred within two weeks of surgery. Follow-up data: Persistence of hiatal hernia on postoperative imaging, the need for an upper airway revision surgery, time to revision surgery, time to first follow-up, and last known observation of associated clinical signs. Data were obtained from medical records.
Main Findings (relevant to PICO question):	Postoperative oxygen: Total of 64/124 received postoperative oxygen support. The use and duration of oxygen support were reported to be dependent on the surgeon’s preference for postoperative care and the technical staff decision to discontinue. 30/64 cases had undergone staphylectomy, while 34/64 cases had undergone folded-flap palatoplasty. Dogs undergoing staphylectomy required a slightly longer median duration of oxygen use. Hospitalisation: The median length of hospitalisation was 1 day for both soft palate procedures (P = 0.94). Minor complications: 27/124 were reported to have developed postoperative regurgitation. 9/124 were reported to have developed aspiration pneumonia. The occurrence of both complications was not different between both soft palate procedures. Major complications: 5/124 were reported to have major complications. Two dogs were euthanised within two weeks of surgery, one underwent a tracheostomy procedure postoperatively, and two underwent a tracheostomy procedure postoperatively and were euthanised within two weeks. Tracheostomy was performed on dogs who could not be extubated, or secondary to severe respiratory distress. Euthanasia was due to persistent severe respiratory distress postoperatively. There was a relatively even distribution of major complications between both surgery types. Follow-up: Of the 14 dogs with evidence of preoperative imaging, only 5 underwent postoperative imaging. 3/3 dogs with persistent hiatal hernias on preoperative and postoperative imaging underwent folded-flap palatoplasty. Two dogs that had staphylectomy procedure had evidence of hiatal hernia on preoperative imaging but this was not noted on postoperative imaging. 7/124 dogs underwent revision soft palate surgeries. Three of them had staphylectomy initially, while four had folded-flap palatoplasty initially.
Limitations:	Due to the retrospective nature of the study, dogs could not be randomised. Soft palate thickness was not measured preoperatively. The surgical procedure and postoperative treatments were determined according to the individual surgeon’s preference. Staphylectomy was only performed using cut-and-sew technique. Vessel-sealing devices, electrocautery or laser were not used which could have affected the outcome. There was a small sample size for some variables such as preoperative and postoperative imaging, postoperative clinical signs, and revision surgeries. 9 dogs who had evidence of hiatal hernias preoperatively did not receive postoperative imaging. The particular type of revision soft palate surgery could not be assessed because only 7 dogs underwent revision soft palate surgery. There was a limited follow-up time with a large range reported.

## Appraisal, application and reflection

Elongated soft palate is a primary component of brachycephalic obstructive airway syndrome (BOAS). Other primary components include stenotic nares, hypoplastic trachea, excessive pharyngeal tissue, and aberrant turbinates (Reiter & Holt, 2018; Oechtering et al., 2007).

Various techniques have been described for the resection of elongated soft palate. Conventional techniques relies on resection of the caudal aspect of the elongated soft palate (staphylectomy) using scissors, laser, or vessel-sealing devices (Brdecka et al., 2008; Riecks et al., 2007). It was reported that these techniques only address the laryngeal obstruction caused by the elongated soft palate but are unlikely to achieve relief of the nasopharyngeal and oropharyngeal obstructions due to thickness of the soft palate. Folded-flap palatoplasty was recommended as a safe and effective technique for excessively thick and elongated soft palate. The folded-flap palatoplasty technique reduces the thickness of the soft palate by removing the majority of the connective and muscular tissues responsible for its excessive thickness, thereby alleviating obstructions in both the oropharynx and nasopharynx. Additionally, the surgical site is positioned rostrally, which ensures that any postoperative swelling or bleeding occurs away from the pharynx, reducing the risk of airway obstruction postoperatively. However, folded-flap palatoplasty has been reported to involve more tissue manipulation and longer surgical times (Findji & Dupré, 2008). Furthermore, a recent study showed frequency of wound healing complications as high as 36% (9/25) following folded-flap palatoplasty (Khoo et al., 2022). However, despite the high complication rate in that study, no associated worsening of clinical signs was observed in the affected dogs.

In a study involving 62 cases, surgical intervention demonstrated an overall treatment success rate of 94.2% (32/34) based on owner assessment conducted over a year after surgery (Riecks et al., 2007). Liu et al. (2017) used exercise tolerance test and whole-body barometric plethysmography (WBBP) as noninvasive tools to provide robust, objective assessments of respiratory function in brachycephalic dogs postoperatively. The study concluded that while the respiratory function of affected dogs improved following intervention, it remained compromised in 68% (34/50) of cases, necessitating regular reevaluations. Another review indicated that advancements in surgical techniques and postoperative care have greatly improved the prognosis of BOAS surgery, even in middle-aged dogs (Dupré & Heidenreich, 2016). Nevertheless, clinicians need to remain vigilant in monitoring and addressing potential complications. During the perioperative phase, clinicians may encounter complications such as aspiration pneumonia, dehiscence at the surgical site, dyspnoea due to inflammation or oedema, and even death (Mercurio, 2011).

The aim of this Knowledge Summary is to evaluate whether one surgical technique for soft palate resection is associated with fewer complications and earlier discharge times. Two recent retrospective cohort studies were found directly comparing staphylectomy with folded-flap palatoplasty and were critically appraised (Fracka et al., 2024; Miller et al., 2024). It is important to note that, although recently described objective outcome measures such as exercise tolerance test or whole-body plethysmography (Liu et al., 2017) were not employed in the evaluated studies, objective parameters, including hospitalisation duration, postoperative oxygen requirement, and incidence of complications, were assessed.

Miller et al. (2024) showed similar anaesthetic, minor, and major complication rates between traditional staphylectomy and folded-flap palatoplasty. The hospitalisation duration was also similar between the two procedures. However, the retrospective nature of the study carries an inherent bias due to the inability to randomise dogs between treatment groups, different preoperative and postoperative diagnostic protocols, lack of standardisation of treatment while in hospital and at discharge, individual surgeon’s preference, and inconsistent follow-up times. The exclusive use of the cut-and-sew technique for staphylectomy limits the assessment of whether alternative techniques could have resulted in more or less morbidity and their potential impact on discharge time. Additionally, the preoperative measurement of the soft palate was not documented, and the presence of a thickened soft palate could potentially influence the choice of surgical procedure. Some variables had a relatively small sample size, which may have contributed to a type II statistical error, potentially influencing the study's findings. Finally, the presence of preoperative clinical signs was relatively limited. This could indicate that the surgical procedure was carried out as a preventive measure. The presence of more severe preoperative clinical signs might impact the rates of intraoperative and postoperative complications and could influence the duration of hospitalisation.

Fracka et al. (2024) identified staphylectomy as one of four variables associated with complicated recovery in dogs undergoing upper airway surgery. Other factors included increasing age, a higher grade of laryngeal collapse, and longer durations of general anaesthesia. However, like the first study, this study was also retrospective, carrying the same inherent limitations. A small sample size and the low incidence of complicated recovery may have introduced an upward bias, requiring cautious interpretation of the results. Additionally, the folded-flap palatoplasty procedure was not standardised, as it involved a mix of sharp dissection, CO_2_ laser, and bipolar cautery techniques.

A possible explanation for the low incidence of complicated recovery with folded-flap palatoplasty in the study performed by Fracka et al. (2024) is that, although staphylectomy is technically easier, takes less time, and requires minimal tissue manipulation (Davidson et al., 2001), folded-flap palatoplasty removes excessive redundant soft tissue from both the larynx and pharynx, relieving airway obstruction more effectively. Folded-flap palatoplasty also reduces the thickness of the soft palate, unlike staphylectomy, which only addresses laryngeal obstruction and does not alter soft palate thickness, causing incomplete relief of clinical signs of obstruction in some patients. Additionally, by moving the surgical site rostrally, folded-flap palatoplasty helps reduce the impact of pharyngeal-laryngeal oedema, thereby lowering the risk of airway obstruction postoperatively (Findji & Dupré, 2008).

Robust objective outcome measures, such as exercise tolerance tests or whole-body plethysmography, were not utilised in the evaluated studies (Fracka et al., 2024; Miller et al., 2024). However, objective parameters such as hospitalisation time, duration of postoperative oxygen requirement, and incidence of complications were evaluated.

In conclusion, the current evidence supporting the superiority of folded-flap palatoplasty over traditional staphylectomy in dogs undergoing BOAS surgery is weak. Both techniques can potentially be used for elongated soft palate surgery with good outcomes. However, the choice of surgical technique should be tailored to the individual patient, taking into account factors such as age, the presence and severity of comorbidities like laryngeal collapse, and the expected surgical time. Further prospective studies are needed to assess whether a specific technique can result in less complications and earlier discharge times following BOAS surgery.

## Methodology

**Search Strategy:** 

Databases searched and dates covered:	CAB Abstracts on OVID Platform [1973–Week 35 2024] PubMed accessed via the NCBI website [1920–September 2024]
Search strategy:	CAB Abstracts: (dog or dogs or canine or canines).mp. (brachycephalic or brachycephalics or BOAS).mp. (palatoplasty or 'folded flap' or folded-flap).mp. (staphylectomy or resection or ‘sharp dissection’ or cutting or 'sealing device' or ((carbon dioxide or co2) and laser)).mp. 1 and 2 and 3 and 4 PubMed: (dog OR dogs OR canine OR canines) (brachycephalic OR brachycephalics OR BOAS) (palatoplasty OR 'folded flap' OR folded-flap) (staphylectomy OR resection OR ‘sharp dissection’ OR cutting OR 'sealing device' OR ((carbon dioxide OR co2) AND laser)) 1 AND 2 AND 3 AND 4
Dates searches performed:	6 Sep 2024

**Exclusion / Inclusion Criteria:** 

Exclusion:	Not relevant to PICO, case reports, review articles, comparing only staphylectomy techniques without folded-flap palatoplasty.
Inclusion:	Relevant to PICO, English language, has at least one technique of staphylectomy compared to folded-flap palatoplasty.

**Search Outcome:** 

Database	Number of results	Excluded – not relevant to the PICO	Excluded – case report	Excluded – review article	Excluded – comparing only staphylectomy techniques without FFP	Total relevant papers
CAB Abstracts	8	2	1	1	2	2
PubMed	8	4	0	0	2	2
Total relevant papers when duplicates removed						2

## ORCiD

Mohamed Sherif: https://orcid.org/0009-0005-1168-1379

## Conflict of Interest

The author declares no conflicts of interest.
